# Treatment of Degenerative Lumbar Spondylolisthesis by Tongdu Bushen Acupuncture, Intradermal Acupuncture, and Moxibustion Integrated Therapy Combined with Core Muscle Training Program: Study Protocol for a Randomized Controlled Clinical Trial

**DOI:** 10.1155/2022/6016542

**Published:** 2022-03-23

**Authors:** Xiaolei Song, Shikui Qi, Jing Gao, Xiaodi Ruan, Shuai Yin, Mingli Wu, Wenbin Fu

**Affiliations:** ^1^The Second Clinical College, Guangzhou University of Chinese Medicine, Guangzhou 510405, China; ^2^Rehabilitation Center, The First Affiliated Hospital of Henan University of Chinese Medicine, Zhengzhou 450000, China; ^3^Henan University of Chinese Medicine, Zhengzhou 450000, China

## Abstract

**Background:**

Degenerative lumbar spondylolisthesis (DLS), one of the most common causes of low back pain, is defined as the displacement of a vertebral body over the lower vertebral body with degenerative changes and the absence of fracture or defect in the vertebral ring. This disease is a common and frequently occurring disease. Currently, there are many clinical treatment methods, but there is no specific method, and most of them have the characteristics of slow effect and easy recurrence.

**Objective:**

The objective of this study is to summarize and analyze the effects of treatment of degenerative lumbar spondylolisthesis by Tongdu Bushen acupuncture, intradermal acupuncture, and moxibustion integrated therapy combined with core muscle training program (CMT) on the improvement of pain degree and dysfunction index, as well as the gait characteristics.

**Methods:**

120 patients with DLS will be recruited and randomly divided into two groups using electroacupuncture combined with the CMT group as the control group. Visual Analogue Scale (VAS), Oswestry Disability Index (ODI), and Japanese Orthopedic Association score (JOA) will be used for evaluation. The spatiotemporal and kinematics parameters in gait analysis, as well as the ability of lumbar muscle contraction, fatigue resistance, relaxation, and coordination of lumbar muscle in surface electromyography (sEMG), will be used as objective observation indexes to observe the effectiveness of Tongdu Bushen acupuncture, intradermal acupuncture, and moxibustion integrated therapy combined with CMT. *Discussion*. Tongdu Bushen acupuncture, intradermal acupuncture, and moxibustion integrated therapy is a traditional Chinese medicine treatment for DLS. Our results will provide evidence to determine whether the integrated therapy can effectively treat DLS, as well as its advantages and safety, and lay a foundation for further research. This trial is registered with ChiCTR2100050409.

## 1. Introduction

Degenerative lumbar spondylolisthesis (DLS), one of the most common causes of low back pain, is defined as the displacement of a vertebral body over the lower vertebral body with degenerative changes and the absence of fracture or defect in the vertebral ring. DLS often occurs at the level of L4. It will cause intervertebral foramen stenosis, compression of nerve roots, and sciatica in severe cases [1]. Studies have shown that the incidence rate of DLS is between 19.1% and 43.1%, with an average age of 71.5 to 75.7 years old, and women are more frequent than men. However, the incidence rate of this disease varies from the crowd and the type of measurement [2].

Although the pathophysiology of the disease has a clearer understanding and there are many clinical treatment methods for DLS, the optimal treatment for this disease is still controversial. For patients with severe DLS, most of them choose the surgical intervention of single decompression or fusion decompression [3] if they have acute spinal cord compression symptoms such as intestinal/bladder dysfunction. Mild DLS patients will choose conservative treatments generally [4], such as activity restriction, the use of anti-inflammatory drugs and epidural steroid injections for pain relief, and other physical rehabilitation exercises [5–8]. For example, exercise therapy is widely recognized as an effective treatment for pain caused by spondylolisthesis [9]. However, due to the lack of prospective studies, the best conservative treatment mode has not been clarified, and most of them have problems such as slow efficacy and easy recurrence.

Studies have shown that acupuncture is an effective way to treat diseases of the lumbar spine [10]. The Tongdu Bushen acupuncture, intradermal acupuncture, and moxibustion integrated therapy is a traditional Chinese medicine therapy for DLS. In addition, the effect of core muscle training program (CMT) on trunk-pelvis kinematics during DLS and healthy gait [11], it also helps to improve the clinical symptoms of patients with DLS. Studies have shown that core stabilization increases the range of motion in the lumbar spine and reduces pain and dysfunction [12]. Compared with conventional physical therapy, the incidence of disability was significantly reduced in patients who received CMT [13]. In order to judge the effectiveness of Tongdu Bushen acupuncture, intradermal acupuncture, and moxibustion integrated therapy combined with CMT in the treatment of DLS, we started this study with electroacupuncture (EA) combined with CMT as the control group. This study is a randomized controlled study. We hypothesized that the method of the experimental group is a more effective method for DLS.

## 2. Materials and Methods

### 2.1. Study Design

**Figure 1:**
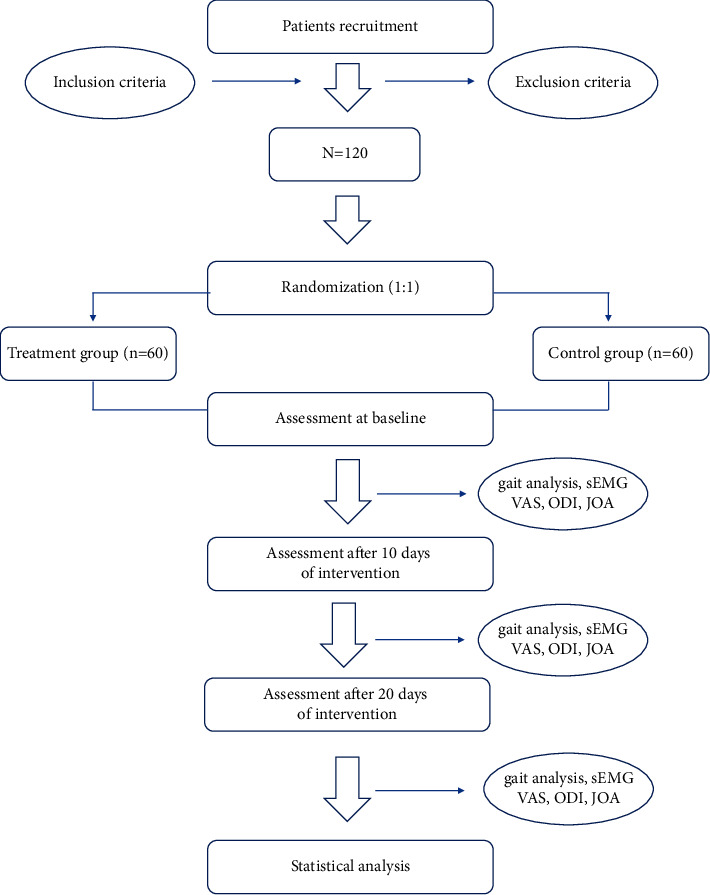
Flowchart of the trial.

This study was designed as a randomized controlled study. Once a treatment has been established as an effective method, it would be unethical to undertake placebo-controlled trials [14], which has led to more widespread application of clinical noninferiority trials over recent decades [15, 16]. A noninferiority trial design could be a better alternative to indirectly show the efficacy of a new treatment [17]. The purpose of this study was to summarize and analyze the effects of treatment of degenerative lumbar spondylolisthesis by Tongdu Bushen acupuncture, intradermal acupuncture, and moxibustion integrated therapy combined with CMT on the improvement of pain degree and dysfunction index, as well as the gait characteristics. Therefore, this study will investigate the efficacy of the experimental group and the control group in the treatment of DLS. In this study, 120 patients with DLS I° who met the criteria of this study will be recruited and randomly divided into the experimental group and the control group, with 60 patients in each group. All subjects will receive a complete written explanation of the study protocol and informed consent. The study flow chart is shown in Figure 1.

### 2.2. Inclusion Criteria

The inclusion criteria are as follows:

1. Meeting the diagnostic criteria for DLS (I° and posterior spondylolisthesis)
2. Adults (50 years ≤ aged ≤75 years) with DLS
3. Having persistent symptoms for less than 24  months before enrollment
4. Patients who had not taken psychotropic drugs before enrollment
5. Patients who had not received traditional Chinese and western medicine for DLS in the first 2 months
6. There were no pathological features that had influence on the results of this study, such as the history of severe lumbar trauma, tuberculosis, tumor, fracture, moderate and severe osteoporosis or above, and other lumbar bone destruction
7. Being willing to give informed consent and cooperate with the subjects of the study

### 2.3. Exclusion Criteria

The exclusion criteria are as follows:

1. Adults (aged ≥75 years or aged ≤50 years) with DLS
2. Spinal cord compression or obvious nerve root injury with low back pain and severe sensorimotor abnormalities of lower limbs
3. DLS with severe blood diseases, hypertension, diabetes, and endocrine system diseases
4. DLS with mental disorders or patients who cannot cooperate with the test
5. Previous lumbar surgery history
6. DLS with severe infection, tumor, osteoporosis, fracture, structural deformity, nerve root syndrome, cauda equina syndrome, and other diagnosed definite pathological changes
7. Patients who took combination medication or participated in other trials during treatment

### 2.4. Sample Size Calculation

This is a randomized controlled trial design, and the primary outcome was that patients' lower back pain improved. Our early clinical observation with a small sample (10 cases) found that 10 days of the experimental group and the control group can reduce scores on the JOA scale by 3.41 ± 1.86 and 2.24 ± 1.67 points, respectively. When the significance test level is set to 0.05 and the test power is set to 0.9, the sample size is calculated as follows:

(1) $$\begin{array}{c} N=2\times {\left[\frac{\left(Z_{\alpha /2}+Z_{\beta }\right)\times \sigma }{\delta }\right]}^{2}. \end{array}$$

When *α* is 0.05 and *β* = 0.1, the normal distribution quantile table shows the following:

(2) $$\begin{array}{c} Z_{\alpha /2}=1.96,\\ Z_{\beta }=1.282, \end{array}$$

where *σ* and *δ* represent the population standard deviation and allowable error, which are 1.86 and 1.17, respectively. The result is 54 by plugging these data into the formula, so 54 patients will be needed in each group. Since there may be a 10% dropout rate, we will recruit 60 participants in each group for this study.

### 2.5. Participants

The study will be conducted at the First Affiliated Hospital of Henan University of Traditional Chinese Medicine (TCM). These DLS patients who meet the inclusion criteria and the exclusion criteria will be recruited through the rehabilitation outpatient or inpatient department of the hospital, the WeChat public account of the rehabilitation center, and the brochure. Recruitment began in June 2021 and is scheduled to end in December 2022.

### 2.6. Randomization

All included participants will be randomly assigned to the experimental group and the control group at a ratio of 1 : 1. This study will be randomized to generate a sequence of random numbers using randomization software, and a specialized statistician who is not a searcher in this study will be responsible for processing the results. Meanwhile, sequentially numbered and sealed opaque envelopes which reveal random numbers will be provided to the patients according to the order of the subjects' visits (the order of visits was consistent with the envelope number), and these envelopes will be hidden until informed consent is obtained.

### 2.7. Blinding

Due to the limitations of acupuncture treatment, the acupuncturists cannot be blinded to who will be informed of the grouping of participants before treatment. The outcome evaluators and data statisticians will be blinded to the group allocation to improve the quality of this study as much as possible. In addition, participants will be assigned to different rooms during the treatment to avoid exchange from each other. Therapists, data managers, and statisticians are not allowed to communicate with others about the treatment of patients.

### 2.8. Interventions

During the following treatment period, all eligible patients will be treated in the assigned groups. The patients in the experimental group will be treated with Tongdu Bushen acupuncture, intradermal acupuncture, and moxibustion integrated therapy, as well as CMT, while patients in the control group will receive EA and CMT. The two groups were assigned their own treatment regimens, treatment once a day, 10 times as a session, for 2 sessions over 3 weeks. (The locations are specifically presented in Table 1). The treatment will be conducted in the Rehabilitation Center of the First Affiliated Hospital of Henan University of Chinese Medicine by professional acupuncturists.

#### 2.8.1. Tongdu Bushen Acupuncture

The traditional chinese medicine believes that kidney essence deficiency and Du Yang indolence are the internal cause of spinal disease. Tongdu Bushen acupuncture mainly involves dredging governor's arteries, promoting blood circulation and collcollars, tonifying liver and kidney, and nourishing qi. The acupoints will include bilateral Shenshu (BL23), Jiaji (EX-B2), Yaoyangguan (GV3), Mingmen (GV4), Yaoshu (GV2), Ashi point (the point where the patient feels most pain), and affected side Huantiao (GB30), Chengshan (BL57), and Weizhong (BL40). First, the skin will be disinfected, and then, single-use acupuncture needles (0.25 ^*∗*^ 40 mm, Hwato, Suzhou, China) will be inserted into acupoints. Acupuncturists will manually stimulate the needles to achieve Deqi. Subsequently, Shenshu (BL23), Mingmen (GV4), Yayangguan (GV3), and Ashi point will be getting moxibustion after acupuncture, and the other acupuncture sites will be irradiated by TDP. The needles will be left for 30 min.

#### 2.8.2. Intradermal Acupuncture

The acupoints will include Ashi point (the point where the patient feels most pain) and bilateral Shenshu (BL23), Yaoyangguan (GV3), Zhibian (BL54), Fengshi (GB31), Yanglingquan (GB34). First, the skin will be disinfected, and then, the intradermal acupuncture is secured to the skin with tape. The needles will be left for 24 h.

#### 2.8.3. Moxibustion

Moxibustion will be carried out along the first lateral line of the bladder meridian. Drugs of moxibustion will include clove, cinnamon, making aconite, asarum, Weilingxian, transbone grass, 120g in total. During the procedure, the patient was placed in the prone position, the back was exposed, and the skin was routinely disinfected with 75% ethanol. Break ginger and filter off part of the ginger juice, stir it evenly with the above powder, and make a rectangular medicine cake about 15 cm wide and 4 cm thick, and lay it flat on the above moxibustion site. Aged fine moxa was selected to make a three-sided moxa pillar with a bottom width of about 5 cm and a height of about 3 cm and placed on the cake and lit moxa. When moxibustion burned at a height of 2/3, the ash was removed, and moxibustion was continued with moxibustion added with moxibustion for 3 times. To the degree that the patient feels warm but can tolerate, the skin at the moxibustion site is slightly flushed after moxibustion, pay attention to prevent scalding, remove the medicine cake and moxa ash after moxibustion, and wipe the back clean with a hot damp towel.

#### 2.8.4. Electropuncture (EA)

The acupoints will include bilateral Yaoyangguan (GV3), Mingmen (GV4), Yaoshu (GV2), and affected side Huantiao (GB30), Chengshan (BL57), Weizhong (BL40). First, the skin will be disinfected, and then, single-use acupuncture needles (0.25 ^*∗*^ 40 mm, Hwato, Suzhou, China) will be inserted into acupoints. Acupuncturists will manually stimulate the needles to achieve Deqi. After feeling of Deqi (the sensations including soreness, numbness, distention, or heaviness), it was connected to the EA device (G6805-2A, Shanghai Huayi Medical Instrument Co., Ltd., China, dense waves 20 times/second, frequency 2.5 Hz). The treatment will be left for 30 min.

#### 2.8.5. Core Muscle Training Program (CMT)

Muscles, such as the multifidus, gluteus medius, transverse abdominis, and internal and external oblique muscles, will be focused on training by the Neurac training device from the Norwegian company Redcord. Breathe evenly during training, and the training will be left for 30 min.

### 2.9. Outcome Measures

Patients will be evaluated with visual analogue scale (VAS), Oswestry Disability Index (ODI), Japanese Orthopedic Association score (JOA), gait analysis, and surface electromyography (sEMG). These indicators can help us to observe the effectiveness of the Tongdu Bushen acupuncture, intradermal acupuncture, and moxibustion integrated therapy combined with CMT in the treatment of pain and physical disorders and provide a theoretical and experimental basis for clinical application.

#### 2.9.1. Primary Index

*(1) Japanese Orthopedic Association Score (JOA)*. JOA is used to evaluate functional disorders [18]. The highest JOA score was 29, and the lowest was 0. Lower scores indicate greater dysfunction. The improvement index, the difference between posttreatment and pretreatment scores, can reflect the improvement of lumbar function before and after treatment. The clinical effect can be understood through the improvement rate (improvement rate is the ratio of posttreatment score minus pretreatment score to 29 minus pretreatment score). The improvement rate can also correspond to the commonly used therapeutic effect criteria: the improvement rate of 100% is cured, the improvement rate of more than 60% is effective, 25–60% is effective, and the improvement rate of less than 25% is ineffective.

*(2) Visual Analogue Scale (VAS)*. VAS is widely used in clinical pain assessment because of its simplicity and practicability [19]. The same method before and after clinical treatment can be used to objectively score and evaluate the effect of pain treatment. Participants looked at a walking scale and said a number between 0 (painless) and 10 (the worst pain to endure) instead of filling out a complex questionnaire. This method is simple, objective, and sensitive.

*(3) Oswestry Disability Index Score (ODI)*. ODI is often used to evaluate low back pain dysfunction [20]. The ODI scale consists of 10 parts, covering pain levels, personal care, lifting objects, walking, sitting, standing, sleep, sex life, social activities, and travel. The scores for each of the above 10 sections range from 0 to 5, with a maximum score of 50. The ODI index was obtained by dividing the patient's actual score by 50 and multiplying by 100%. The higher the ODI index, the more serious the patient's dysfunction.

#### 2.9.2. Secondary Indicators

*(1) Gait Analysis*. Recent studies have shown that DLS can affect patients' gait, which can reflect the functional activities of musculoskeletal muscle in patients with DLS. At present, 3D gait analysis has been used in the diagnosis, treatment, and rehabilitation of DLS. Previous studies have systematically studied the gait of DLS patients from the perspectives of spatiotemporal parameters, kinematics parameters, dynamics parameters, and surface electromyography. It is found that the abnormal movement mode of muscles may be the cause of the decline of hip and knee joint range of motion and lumbar compensatory movement in DLS patients during walking.

*(2) sEMG*. Muscles play an important role in overall dynamic stability during functional tasks. sEMG provides an effective method to evaluate muscle response, such as detecting the changes of trunk muscle activity during walking. sEMG records the neural electrical signals received by the muscle and reflects the activity state of the muscle to some extent through the amplitude and activation time of the electrical signals.

### 2.10. Data Management and Monitoring

Case report forms (CRF) include observation points, treatment points, outcome measures, adverse events, and safety assessments. The researchers will be required to fill in the relevant information in a timely and accurate manner according to the requirements of the CRF. All data obtained will be kept strictly confidential and will be stored electronically in a secured and restricted-access database. Participants will be randomly numbered, and other personally identifiable information will be removed.

### 2.11. Statistical Analysis

All statistical analyses will be performed using SPSS Statistics for Windows Version 23.0 (IBM Corp., Armonk, NY). The normality test will be applied to all data. The *t*-test will be used if the data fit the normal distribution; otherwise, the rank sum test will be used. Counting data will be compared using the chi-square test. The significance level will be set at *P* < 0.05 and the confidence interval at 95%.

### 2.12. Withdrawal and Dropout

The subject will be excluded from the study if the subject does not meet the inclusion and exclusion criteria or withdraws his/her consent, or the subject's continued participation is judged as inappropriate. The researchers will record the reason for any interruption in the intervention and whether each participant completed the study.

### 2.13. Safety

The occurrence of all adverse events will be assessed at each visit. Subjects will be monitored for undesirable, unintended symptoms, signs, and illnesses. The number and percentage of subjects who experience at least 1 adverse event will be calculated. To ensure the safety of participants, the data collection process will be supervised by the project leader. At each visit, participants will be required to stay in the hospital for 30 minutes after treatment and will be asked about any adverse events during the study that showed signs of acute adverse events. Adverse effects will be recorded at each visit during treatment.

### 2.14. Ethics

This study is designed based on the principles of the Helsinki declaration and has been approved by the Ethics Committee of the First Affiliated Hospital of Henan University of Chinese Medicine (2021HL-192). After careful discussion and modification by the project team, the protocol is registered on the website of the Chinese Clinical Trial Registry (ChiCTR) (ChiCTR2100050409). Participants will be informed of the possible risks and other related matters of the study protocol before entering the study and will sign an informed consent before randomization. All participants of this study will be allowed to withdraw their consent at any time for any reason.

### 2.15. Quality Control

Unified training will be carried out so that participating doctors, nurses, and jurors can fully understand the whole trial process before the clinical trial. To guarantee the quality of the whole trial, two supervisors will be sent to assure whether (1) all participants meet the inclusion criteria and do not meet the exclusion criteria, (2) all the participants fully follow the clinical trial process, and the CRF has been completed. The standard operating procedures (SOP) will be invariably followed. Drop-outs, withdrawals (and the reasons), and any compliance of all patients occurring will be recorded in detail by the inspectors throughout the treatment and follow-up period.

## 3. Discussion

DLS is a common disease of the spine. At present, the treatment methods of DLS are mainly surgical treatment and conservative treatment. It has been shown in several studies, the risk of iatrogenic injury and the development of lower extremity deep vein thrombosis or pulmonary embolism may occur in patients with DLS after decompression surgery [21–24]. Therefore, conservative treatment of DLS is preferred [4]. The decompression surgery is performed only on the patient who was lumbar spondylolisthesis combined with neurological symptoms of the lower extremities and severe lumbar spondylolisthesis that cannot be treated conservatively [25]. It has been shown that acupuncture and CMT are more effective than analgesics or nonsteroidal anti-inflammatory drugs (NSAIDs) [10, 13]. As DLS is a chronic disease, most people take NSAIDs for a long time, which often causes gastrointestinal side effects and nephrotoxicity [26, 27].

Acupuncture, as a traditional treatment in Chinese medicine, has shown its efficacy in alleviating DLS symptoms. This study is designed to focus on the treatment of DLS and introduce modern medical research methods such as sEMG and gait characteristic analysis, which provides a methodological reference for the mechanism study of traditional Chinese medicine and is of great significance to reveal the multitarget effect of acupuncture. Our study is the first elaborately designed, randomized controlled trial to our best knowledge. It will provide an option of acupuncture treatment for the DLS patients and physicians as a better disease remission if this trial succeeds.

Hopefully, this trial will produce high-quality evidence pertaining to the efficacy and safety of Tongdu Bushen acupuncture, intradermal acupuncture, and moxibustion integrated therapy combined with CMT in treating DLS. The results will aid in clinical decision-making for the management of symptomatic DLS and provide useful information that can be incorporated into future guidelines.

## Figures and Tables

**Table 1:** Locations of acupoints for acupuncture.

Chengshan (BL57)	In the posterior area of the calf, the gastrocnemius muscle and the tendon intersection
Zhibian (BL54)	In the sacral area, the fourth posterior sacral foramen was horizontally equated, and the middle sacral crest was 3 inches apart
Weizhong (BL40)	In the posterior area of the knee, midpoint of the popliteal stripe
Shenshu (BL23)	In the spinal region, below the spinous process of the second lumbar vertebra (L2), 1.5 inches is opened beside the posterior midline
Jiaji (EX-B2)	In the spinal region, after the midline side open 0.5 inches, 17 points on one side from the first thoracic vertebra (T1) to the fifth lumbar vertebra (L5) on both sides
Yanglingquan (GB34)	In the anterior and lower depression of the fibula head on the outside of the calf
Fengshi (GB31)	In the iliotibial band, the middle fingertip in the depression when the hand is upright and the palm is against the thigh
Huantiao (GB30)	In the gluteal region, the outer 1/3 and inner 2/3 of the junction between the most convex point of the greater trochanter of femur and the sacral hiatus intersect
Mingmen (GV4)	In the depression inferior to the spinous process of the second lumbar vertebra (L2), on the posterior median line
Yaoyangguan (GV3)	In the depression inferior to the spinous process of the fourth lumbar vertebra (L4), on the posterior median line
Yaoshu (GV2)	In the sacral region, directly opposite the sacral hiatus, posterior midline

## Data Availability

No data were used to support this study.

## Abbreviations

DLS: Degenerative lumbar spondylolisthesis
EA: Electropuncture
CMT: Core muscle training program
VAS: Visual analogue scale
ODI: Oswestry disability index score
JOA: Japanese orthopedic association score
sEMG: Surface electromyography
CRF: Case report forms.

## Supplementary Materials

Supplementary Materials Additional file 1: Completed Standard Protocol Items: Recommendation for Interventional Trials (SPIRIT) 2013 Checklist: items addressed in this clinical trial protocol.
